# Cost analysis of a teaching hospital in Thailand: Impacts of the first wave of COVID-19

**DOI:** 10.1371/journal.pone.0273771

**Published:** 2022-09-01

**Authors:** Nopphol Witvorapong, Sureerat Ngamkiatphaisan, Jiruth Sriratanaban

**Affiliations:** 1 Center of Excellence for Health Economics, Faculty of Economics, Chulalongkorn University, Bangkok, Thailand; 2 Data Management and Cost Evaluating Center, King Chulalongkorn Memorial Hospital, Bangkok, Thailand; 3 Department of Preventive and Social Medicine and Research Center for Health Systems and Services, Faculty of Medicine, Chulalongkorn University, Bangkok, Thailand; Syreon Research Institute, HUNGARY

## Abstract

COVID-19 has had adverse impacts on the health sector in Thailand and information on hospital costs is required for planning and budgeting. The aim of this study was to estimate costs that the pandemic imposed on a teaching hospital in the country, focusing on the first wave which took place in March-May 2020. A retrospective cost analysis was performed. Data on COVID-related activities, including when and where they were undertaken, were retrieved from existing sources and supplemented by in-depth interviews with the hospital’s staff. The data collection period was January-October 2020, covering three distinct phases: before, during, and after the first wave of the pandemic. The total costs during the preparation phase in January-February, the pandemic phase in March-May, and the standby phase in June-October were 0.6, 3.9, and 1.2 million US dollars respectively. Costs related to treatment of COVID-19 patients were higher than those related to infection control in the first two phases but not in the standby phase, making up 82.09%, 75.23%, and 43.95% of the total costs in the three phases respectively. Costs were incurred in all areas of the hospital, including those that were set up to serve COVID patients, those serving non-COVID patients, and those serving both groups. Public donations were integral to the provision of services and made up 20.94% of the total cost during the pandemic phase. This study was the first to estimate hospital costs of COVID-19 in Thailand. It demonstrated high costs of a national outbreak and supported the establishment of a contingency fund for medical emergencies at the hospital level.

## Introduction

With the first confirmed case in January 2020, Thailand became the first country to report a case of coronavirus outside of China. The first wave of the pandemic began in March 2020 and ended in late May, triggered by imported cases and clusters of bar patrons and people participating at a boxing event [1]. The second wave emerged in December 2020 and ended by February 2021, caused by illegal cross-border movements of Thai and migrant workers [2]. The third wave hit the country in April—June 2021, starting at affluent entertainment districts in Bangkok before trickling down to other areas. The fourth wave in July—December 2021 witnessed a surge in the infection rate in light of the Delta variant [3]. As of August 2022, the country is in the midst of the sixth wave, with the Omicron variant as the dominant strain. According to the Ministry of Public Health, the daily case count has been at the 5-digit level since February 2022 and the total number of COVID cases since its inception currently stands at about 4.6 million and the number of deaths is close to 32,000.

The government has put in place a system to curb the spread of the pandemic. Established in March 2020, the Center for COVID-19 Situation Administration (CCSA) is chaired by the Prime Minister, serves as the headquarters for COVID-related policies, and is responsible for coming up with mitigation measures, coordinating among agencies in the implementation of such measures, and handling public communications [1, 2]. Both social and public health measures have been used. The former includes, for example, work-from-home orders, a temporary closure of non-essential businesses, and the imposition of national curfews [1]. The latter refers to health-system responses and encompasses case investigation, contact tracing, case surveillance at the community level, treatment, and isolation in quarantine sites [2], with additional home-based isolation measures from the third wave onward. These measures are supplemented by constant supply-side expansion efforts by the government, including a scaling up of real-time PCR laboratory capacity, the installation of negative pressure rooms in selected hospitals, the construction of quarantine sites, and the acquisition of additional personal protective equipment and medicines [2].

With the presence of universal health coverage, a strong public health system, and centralized planning under the CCSA [1, 2, 4], Thailand was able to handle the first and the second waves of the pandemic effectively. In the first wave, the total number of cases was 3,042 with 57 deaths, and there was no local transmission from late May until the end of 2020 [1, 2]. Despite the surge in the number of cases (approximately seven times higher than the first wave), the second wave was contained in only two months, as a result of targeted lockdown measures and active case finding [2]. However, the country has struggled since. With public pressures for economic activities to resume, social measures have been considerably relaxed. The predominance of the Delta and subsequently the Omicron variants with increased transmissibility also means that it has become easier for people to contract and spread COVID-19 to others.

The pandemic has exerted not only clinical pressure on the health system but also financial pressure [5]. According to the 2020–2021 Royal Acts on government borrowing, the Thai government took out a 1.5-trillion Thai Bath (THB) loan (approximately 45 billion US dollars (USD)), from which the Ministry of Public Health received 1,400 million USD in 2020 as a response to the first wave and in anticipation of future waves of COVID-19. Since the budget allocation was based on funding availability and not on costs of COVID-19 case management, it was possible that the budget earmarked for COVID-19 within the health sector was inadequate, imposing undue financial stress on health care providers.

Information on costs of COVID-19 at the hospital level is crucial for effective planning and budgeting. In order to battle the pandemic and prevent a fall of the health care system, health care providers need to be to sufficiently subsidized in order to stay afloat [5]. At least, their costs should be covered. The issue is important for Thailand, whose health service delivery system is characterized by an unbalanced public-private mix, with 80% of the nation’s health care resources (including physicians, hospitals, and hospital beds) belonging in the public sector [1, 4]. As universal health coverage has been achieved, patients are provided with subsidized access to COVID-19 services [2]. Public and private health care providers are differentially reimbursed, based on guidelines set by the Ministry of Public Health and the National Health Security Office (NHSO) [6]. However, it is unclear how the reimbursement rates were set.

In fact, it has been suggested that the reimbursement schedule may be inadequate. Legally required to accept COVID-19 patients under the government’s terms of payment, private hospitals have lodged complaints that the reimbursement rate is lower than their operation costs [7]. Similarly, public hospitals could be adversely affected. Financial sustainability of public hospitals has been a subject of debate in the past two decades [4]; public hospitals are required to serve beneficiaries of all major public health insurance schemes, the most important of which is Universal Coverage Scheme (UCS) covering about 73% of the population [1]. The UCS’s complex provider payment method is based largely on the concept of capitation, where health care providers are given a semi-fixed amount of payment for treating a predetermined quantity of patients with a comprehensive health care benefits package. Under the system, health care providers absorb most of the financial risk [4]; in 2017, according to an unpublished report by the Ministry of Public Health, 211 public hospitals were assessed as being in a critical financial condition. The COVID-19 situation requires that public hospitals perform tasks of outbreak responses in addition to their routine responsibilities [1, 2], which could make them more financially vulnerable without proper compensation.

Despite the importance of information on hospital costs, there have been no COVID-19 costing studies from the provider’s perspective in Thailand to date. The aim of this study was to estimate the financial burden that COVID-19 imposed on a large teaching hospital in Thailand, using data from the first wave as they were sufficiently documented. Results from this study could be used an input into the revision of the COVID-19 reimbursement schedule in Thailand. Also, this study methodologically complemented published works on COVID-19 costing [8–11] and infection control [12–25] in other countries, as it explored the role of public donations and outlined COVID-related activities in a detailed manner across different phases of the pandemic and different areas of the hospital.

## Materials and methods

### Study design

This study performed a retrospective cost analysis of the first wave of COVID-19, using existing data from a large teaching hospital in Thailand. The study was approved by the Faculty of Medicine, Chulalongkorn University, Institutional Review Board (IRB) (COA 846/2020) on 8 July 2020. Informed consent was waived by the IRB as the analysis was performed anonymously, based only on aggregate data, and the authors did not have direct access to personal information of individual patients.

### Study setting

The setting of the study was a public teaching hospital in Thailand. Located in Bangkok, it housed 1,433 beds, was composed of 71 departments, and, in 2019, treated approximately 350,000 outpatients and 50,000 inpatients. It was classified as a ‘super-tertiary’ care center, providing the most advanced level of health services in the country.

The hospital had a complex financial structure. According to its annual report, the hospital received revenue from three major sources. The first source was cash donations, which were seasonal and motivated by the fact that they were tax deductible. The second source was revenue from activities unrelated to direct patient care. They included, for example, renting out space for retail stores, charging parking fees, and sale of over-the-counter medicines. These made up 6.21% of the hospital’s total revenue in the fiscal year of 2020 (excluding cash donations). The final source was hospital charges. Patients were split into (1) cash-pay and privately insured patients, who together contributed approximately 37.83% of the total revenue in 2020 and whose payment was received (almost) immediately after treatment provision and (2) patients with a public health insurance coverage, who contributed about 55.96% of the total revenue in the same fiscal year but whose payment was delayed as their medical bills needed to be audited by relevant agencies before the hospital was reimbursed. The 2020 financial statements showed that the delay could be substantial, lasting up to 15 months, and that it was possible that the reimbursement was lower than the hospital’s actual cost (as discussed in the Introduction [4]). The mismatch between the time at which treatment was provided and when it was reimbursed as well as the potentiality of the reimbursement being lower than costs suggested that the hospital’s revenue was not necessarily an accurate indicator of the hospital’s month-on-month financial health.

Also, the hospital was affiliated with a public university and received resources from them. The university provided free use of the land on which the hospital was situated and, as part of their training, unpaid medical students contributed to the hospital’s operations. This unique relationship had an implication on costing, as discussed below.

### Data collection

This study was conducted in April 2020-May 2021 and collected data regarding the hospital’s operations during January-October 2020, covering the months before, during, and after the first wave of the pandemic. The hospital did not maintain a database specifically designed for cost calculation and also did not have a separate database for COVID-19.

Data were obtained from several existing sources. The first source was the hospital’s annual report and its business continuity plan, which documented COVID-19 treatment procedures, infection control measures, and resource utilization over time. The second source referred to work schedules and duty hours of medical and non-medical personnel. The third source was a database on financial management, encompassing procurement details and information on prices and quantities of medical equipment and materials as well as the date on which each item was acquired. The fourth source included lists of medical supplies, medicines, and medical equipment that were used during the data collection period. The fifth source referred to records of lab tests and chest x-rays. The sixth source was daily financial statement, with information on charges and health services utilization of cash-pay and insured patients. The seventh and final source was the hospital’s Data Center, a division that was responsible for computing annual operation and medical service costs. They provided input prices (e.g., cost of one minute of doctors and depreciation cost and useful life of each building) and records of durable items that had been purchased prior to the pandemic.

Also, semi-structured interviews were conducted in April-August 2020. A total of five interviewees were purposively selected to represent different types of personnel that were involved with the clinical and financial aspects of the hospital’s response to the pandemic. They included the hospital’s management, members of the finance and accounting departments, and medical personnel who worked at a COVID ward. Each interview lasted approximately an hour and the following questions were asked: clinical and financial impacts of the pandemic, the hospital’s COVID-19 response strategy, the extent to which external assistance was required and received, and expected future impacts of COVID-19. These interviews were used to enrich the authors’ understanding of how the hospital handled the pandemic, which led to continual revision of the study design, and to supplement the above data sources, providing an approximation of missing data and a way to triangulate data that were inconsistently documented.

### Cost analysis

Based on the provider’s perspective, this study used a bottom-up costing approach. The process involved several steps. The first step was to identify COVID-related activities, based on a literature review and interviews with the hospital’s staff. The second step was to quantify the amount of resources, including capital, labor, and materials, used for each activity within the data collection period. At this stage, costing assumptions were made. The final step was to embellish the costing framework with information on when and where each of the activities took place, thereby providing time and space dimensions to the costing process (as opposed to producing a single estimate).

Isolating COVID-related activities from other activities within the hospital in order to avoid double counting, this study produced incremental costs, i.e., costs that were incurred due to COVID-19. Resources used directly and solely for the purpose of tackling COVID-19 were separated from those that were used indirectly or not used for COVID-19 at all. For example, while depreciation expense for spaces dedicated for COVID-19 was included as part of the total cost, that for spaces used for infection control was not included. The idea was that the former could have been used for other activities in the absence of COVID-19, yet the latter were used for ongoing non-COVID activities and therefore would have generated costs for the hospital anyway, with or without COVID-19. The same logic applied for all types of resources considered in this study.

#### COVID-related activities

To identify COVID-related activities, the hospital’s reports and existing studies on hospital infection were reviewed [11–25] and their cost items were merged. The review suggested that hospital-based activities in the presence of an infectious disease could be categorized into three groups. The first group referred to activities related to treatment of the disease itself, including diagnostic procedures, treatment procedures, and personnel benefits during the pandemic. The second group referred to measures to minimize intra-hospital infections, which included, for example, access control, postponement of medical appointments, and home delivery of drugs for patients with non-communicable diseases. The third group referred to activities that served the purposes of both treatment and infection control. They included, for example, intra-hospital communications, off-site and on-site screening, and toxic waste management. The threefold classification was verified and activities specific to the hospital within each activity group were laid out during the interview process. The first column of Table 1 shows all cost items included in this study and how they were identified.

**Table 1:** Cost items and COVID-related activities.

Activities [Reference]	Examples of resources used ^a^:			Dimensions	
	Capital	Labor	Materials/ Expenses	Time ^b^	Space ^c^
**Treatment**					
Diagnostic procedures [13–15, 18, 19, 21, 23, 25]	Cost of space used; durable office supplies; acrylic aerosol partitions.	Physicians (number of shifts from Jan-May 2020 respectively = 96; 191; 841; 764; 275), nurses (number of shifts from Jan-May 2020 respectively = 364; 419; 1,845; 2,142; 545); assistant nurses; technicians; non-medical personnel.	Personal protective equipment (PPE); testing solutions; swabs; newly acquired non-durable medical and office supplies.	Prep, P, S	COVID, Semi
Treatment procedures [12–15, 17–19, 21–25]	Cost of space used; cost of setting up negative pressure rooms; biosafety cabinets; medical vacuum pump systems.		PPE; antiviral drugs; newly acquired medical supplies; alcohol solutions.	Prep, P, S	COVID, Semi
Laboratory and X-ray analyses [18, 21, 22]	Unit costs of lab and diagnostic imaging tests were provided by the hospital.			P	COVID
Renovation in COVID and semi-COVID wards [12, 15, 21]	Cost of renovation (e.g., installation of bathrooms for staff, access control and ventilation systems) (externally sourced).	-	Cost of cleaning and moving office and medical supplies to designated space for COVID treatment (externally sourced).	Prep, P, S	COVID, Semi
Post-discharge care [Interviews]	-	Nurses (n = 18) and non-medical personnel (n = 11).	Cost of food and renting a space for patient recovery (known as “Hospitel”).	P	COVID
Room and board for staff [Interviews]	-	Hotel stays for staff unable to go home (n = 25, daily) from 26 March- May; meals for staff (100,000 boxes donated).	-	P	COVID
Life insurance for staff [Interviews]	-	Life insurance premia for staff for one year, from April 2020 (n = 9,083).	-	P, S	COVID
**Infection control**					
Infection control [13, 15, 16, 18, 19, 21–23, 25]	Cost of renovation in surgery rooms; biosafety cabinets; ventilators (CPAP and BiPAP)	-	Use of PPE on non-COVID patients; disposable airway adapters; alcohol solutions.	Prep, P, S	Non, Semi, R
Security and surveillance [12]	Infrared thermometers.	12 temperature screening checkpoints, manned by non-medical personnel (n = 2 per checkpoint, with one shift each daily).	Alcohol gel; face masks.	Prep, P, S	All
Establishment of an acute respiratory illness clinic [Interviews]	Cost of setting up the ARI clinic (including renovation and signage).	-	-	Prep, P, S	Non
Renovation in non-COVID areas [Interviews]	Cost of installing access control and ventilation systems; thermometers; pulse oximeters.	-	Cost of moving supplies; temporary signage to direct foot traffic and separate COVID from non-COVID patients (externally sourced).	Prep, P, S	Non
Postponement of medical appointments [Interviews]	-	Nurses contacting patients to postpone appointments (1 shift, daily).	Cost of calling/texting patients (around 2,500–4,500 patients in April- May 2020), using the cost-per-call rate of 3 THB.	Prep, P	Non
Home delivery of drugs for NCD patients [Interviews]	-	Non-medical personnel packing and posting drugs to patient homes (n = 1, one shift per day).	Cost of mailing drugs to 1,250 patients, using the postage rate of 20 THB (rate for packages < 1 kilogram).	Prep, P, S	Non
**Treatment and infection control**					
Preparation and communications [12, 19, 23–14]	-	-	Meeting expenses, e.g., use of teleconference facilities and snacks during breaks.	Prep, P	R
Administrative costs [18, 21]	-	Overtime of personnel unrelated to direct patient care (Not documented).	-	Prep, P, S	R
Waste management [15, 16, 19, 21–23]	-	-	Cost of (toxic) waste disposal (externally sourced).	P, S	R
Screening: off-site and on-site [15, 16, 18, 19, 21–23, 25]	-	Physicians (n = 3) on an instant messaging ("Chatbot”) system; nurses (n = 8) asking about patients’ travel history.	Cost of acquiring an official account on the instant messaging system.	Prep, P, S	R

^a^ n = number of people involved in the activity

^b^ Prep = Preparation; P = Pandemic; S = Standby

^c^ COVID = COVID areas; Non = Non-COVID areas; Semi = Semi-COVID areas; R = Remaining areas; All = All areas

#### Costing assumptions

Quantities and unit prices of capital, labor, and material inputs for each activity within each month of the data collection period were quantified under a set of assumptions. First, this study included buildings, equipment, and tools as capital items but did not include land. This was based on the fact that the hospital had free use of land under the university’s provisions (as explained above). It was also consistent with previous studies where land was excluded from hospital costs, suggesting that, with an unlimited useful life, land was not a depreciable asset and the determination of its value was outside of hospitals’ control [26, 27]. A straight-line depreciation method was employed to annualize costs of capital items using their respective life years and the salvage value of 1 Thai Baht (THB), which was equivalent to approximately 0.03 US dollars (USD). Annual capital costs were divided by 12 before they were turned into monthly costs. The method was based on costing guidelines set by the Thai Ministry of Finance and Ministry of Public Health.

Second, to compute labor costs for a given activity, salaries, overtime payment, and duty premia of each type of personnel were summed, converted into per-shift costs, and multiplied by the number of shifts spent on the activity. During the pandemic, doctors and nurses were respectively given an ‘incentive’ payment of 1,500 and 1,000 THB per shift from the government. These payments were counted as costs of the pandemic, although the hospital did not bear such costs. Unpaid medical students also provided selected services, most notably, off-site and on-site screening. Their labor was not costed because data regarding their work were inconsistently documented and it was not clear whether their contributions should be considered as part of the medical training or as an input toward the hospital’s response to the pandemic.

Third, material and pharmaceutical costs were based on actual prices and, in cases where prices were undocumented, procurement prices or, secondarily, market prices were used. Expenses for services provided by external organizations, e.g., utilities (including waste disposal), cleaning and moving costs, and room rentals for post-discharge care, were included as part of the material cost.

Finally, some capital items and materials were donated during the pandemic. Market prices were used to estimate their values. Similar to the incentive payments, donated items were considered to be part of COVID-19 costs, although they were not absorbed directly by the hospital. The total cost of each activity in each month was the summation of relevant labor, material, and capital costs. Examples of capital, labor, and material inputs used for each of the COVID-related activities are shown in Columns 2–4 of Table 1.

#### Time and space dimensions of costing

Having investigated the hospital’s operations during COVID-19, this study refined the above costing framework in two important ways. First, instead of estimating only costs incurred during the pandemic, this study demonstrated that resource utilization varied over time. The data collection period purposely covered three phases of pandemic management. January-February 2020 marked the first (‘preparation’) phase. Here, the hospital was notified of a potential respiratory outbreak, generated plans regarding clinical management for ICUs and operating rooms, and repurposed areas in preparation for the outbreak. In this period, the hospital received 88 patients with suspected COVID-19, none of which became confirmed cases. The second (‘pandemic’) phase was in March-May 2020, which also marked the first wave of the pandemic in Thailand [1]. During this phase, the hospital provided testing for the general public and treatment for confirmed cases. Also, to minimize non-essential contact, it closed most clinics and wards, implemented work-from-home initiatives, and offered telemedicine services to patients with chronic diseases. The number of patients who were tested for COVID-19, those under investigation (PUI), and those who were admitted as inpatients at the hospital were 3,137; 1,100, and 419 respectively. The final (‘standby’) phase covered June-October 2020, with October marking the end of the fiscal year. With no domestic cases, most personnel were reallocated to their original departments. Nevertheless, all COVID-19 facilities, inclusive of medical equipment, were maintained as future waves were expected. The phase definition corresponded to details in the hospital’s COVID-19 reports and was verified by informants during the in-depth interviews.

Also, this study performed costing for different areas in the hospital, instead of concentrating on COVID wards. The hospital was divided into four areas. COVID areas referred to wards and clinics dedicated to serving patients who wished to be tested, those with suspected or confirmed COVID-19, and those recovering from the disease. Only treatment costs (and activities) were incurred here. Non-COVID areas referred to spaces in the hospital that served primarily non-COVID patients, e.g., NCD clinics. Only infection control costs (and activities) were incurred here. Semi-COVID areas referred to departments where preparation for potential COVID cases was necessary but whose main purpose was not to serve COVID patients. They included labor and delivery rooms and departments of emergency medicine and laboratory medicine. Finally, there were activities that were part of the hospital’s operations during COVID-19 but did not take place at any of the above area categories specifically. An example was access control, which was undertaken at the entrance of every building. These activities were considered as having taken place at the ‘remaining areas’. In semi-COVID areas and the remaining areas, costs included both treatment and infection control costs. The final list of activities, the area classification, and the costing process were crosschecked and agreed upon by the hospital’s management during the interviews.

The time and space classifications were accounted for in the calculation of costs in this study. They represented methodological contributions beyond what was already discussed in the literature [11–25] and could be generalized to other settings. The final two columns of Table 1 summarize the discussion of the time and space classifications above.

## Results

Table 2 presents costs in US dollars and ratios of different cost items to the total costs for the three phases of COVID-19, split by type of inputs, activity, hospital area, funding source, and costing method, as shown in Panels A-E respectively. In each panel, all items sum up to the total costs. Average monthly costs are also provided.

**Table 2:** Cost items and cost breakdown of the first wave of COVID-19.

Cost items	Study Period		
	Phase 1 ‘Preparation’	Phase 2 ‘Pandemic’	Phase 3 ‘Standby’
**Month**	Jan-Feb 2020	Mar-May 2020	Jun-Oct 2020
**Number of patients**			
PUI	88	1,100	0
Confirmed cases [Average LOS ^a^ = 4.77]	0	419	0
Confirmed cases needing ICU care [Average LOS in the ICU ^a^ = 4.85]	0	22	0
Non-COVID outpatients ^b^	58,333	72,917	145,833
Non-COVID inpatient admissions ^b^	8,333	10,833	20,833
**Panel A Cost by type of inputs** ^c^			
Labor (including incentive payments)	19.77%	29.06%	14.84.%
Material	66.07%	62.29%	23.02%
Capital	14.16%	8.65%	62.15%
**Panel B Cost by activity** ^c^			
Treatment	82.09%	75.23%	43.95%
Infection control	17.91%	24.77%	56.05%
**Panel C Cost by area** ^c^			
COVID	79.07%	55.17%	36.91%
Semi-COVID	5.80%	15.21%	28.74%
Non-COVID	11.20%	15.34%	19.51%
Remaining areas	3.94%	14.28%	14.84%
**Panel D Cost by funding source** ^c^			
Hospital itself	92.75%	79.06%	99.50%
Government (incentive payments)	7.18%	7.51%	0.00%
Public donations	0.07%	13.43%	0.50%
**Panel E Cost by costing method** ^c^			
Direct	81.97%	64.65%	51.28%
Indirect	18.03%	35.35%	48.72%
**Total cost (in USD)** ^d^	605,984.68	3,891,705.94	1,234,424.13
**Average monthly cost (in USD)** ^d^	302,992.34	1,297,235.31	246,884.83

^a^ LOS = length of stay (nights)

^b^ The numbers of non-COVID outpatients and inpatient admissions were approximated, based on the 2019 utilization figures and the hospital’s report on the first wave of COVID-19, which suggested that 50% of outpatient and 40% of inpatient services were reduced for 30 days during April–May 2020.

^c^ Figures in Panels A-E refer to percentages of the total costs.

^d^ Exchange rate of 1 USD: 30 THB was used.

Health care utilization varied throughout the data collection period. Phase 1 had only patients under investigation (PUIs), Phase 2 had 419 confirmed cases, while Phase 3 did not have any COVID patients. All PUIs were asked to stay overnight at the hospital for further observation, following the Ministry of Public Health’s protocol during the first wave of the pandemic. All confirmed cases in Phase 2 were admitted as inpatients and 22 of such cases needed intensive care, having been assessed as having a critical condition. The average lengths of stay for the confirmed cases and the ICU cases were 4.77 and 4.85 nights respectively, and all confirmed cases were transferred to an outside facility for post-discharge care and spent an average of 9.82 nights there. The monthly numbers of non-COVID outpatients and inpatient admissions in Phase 2 were lower than in Phases 1 and 3, as the hospital reduced some of its services during the peak of the first wave.

The total costs in Phase 1, 2 and 3 were 0.61; 3.89; and 1.23 million USD respectively. The average monthly cost during the ‘pandemic’ phase was more than four and five times that of Phase 1 and Phase 3 respectively. Consistent with the fact that the number of COVID-19 patients varied over time, labor costs of the three phases differed. While the majority of labor costs in Phases 1 and 2 were derived from the provision of treatment services by medical staff, the majority of labor costs in Phase 3 were related to personnel benefits (i.e., COVID-19 insurance, which was provided to all staff throughout the fiscal year) and access control, where non-medical staff were assigned to perform temperature checks upon patients entering each building in the hospital. Material costs were the major cost drivers in Phases 1 and 2. They pertained notably to personal protective equipment (PPE), N95 masks, test kits, and laboratory supplies for diagnostic purposes, and also included payments for one-time services performed by outside organizations, e.g., cleaning and moving expenses. In Phase 3, material costs were derived most importantly from continued use of PPE, non-durable medical and office supplies, and alcohol gel. Finally, in all three phases, capital costs included costs of spaces and medical equipment used for COVID-19 activities. Major capital items included the cost of converting existing spaces into COVID wards and screening stations in Phase 1 and the cost of setting up negative pressure rooms in Phase 2. In Phase 3, since the hospital did not dismantle any of the COVID and semi-COVID wards and did not relocate any of the equipment therein, the capital costs in the first two phases were carried over and combined with purchases of additional oxygen devices and infrared thermometers, making the total capital cost in Phase 3 higher than the earlier phases despite the absence of patients.

Results from Panels B and C can be considered together. COVID areas served primarily COVID patients, performing diagnostic and treatment procedures and encompassing expenses related to COVID insurance as well as room and board for medical staff in COVID wards. The entirety of costs incurred here was placed under treatment costs. Non-COVID areas served primarily non-COVID patients and undertook only infection control activities, including, for example, use of PPE and repetition of temperature checks. Costs incurred in non-COVID areas were placed under infection control. Activities observed in both COVID and non-COVID areas took place in semi-COVID areas. Similarly, activities performed in the remaining areas, e.g., intra-hospital meetings and toxic waste management, could fall under either treatment or infection control, serving both COVID and non-COVID patients. Costs in both semi-COVID and the remaining areas were divided into treatment and infection control costs in Phases 1–2, while they were treated only as infection control costs in Phase 3 given the absence of COVID patients. The allocation for Phases 1–2 was based partly on the in-depth interviews and partly on the distribution of COVID vis-à-vis non-COVID patients, whenever such data were available. It turned out that 82.09%, 75.23%, and 43.95% of the total costs were treatment costs in Phases 1–3 respectively. The treatment cost in Phase 3 did not refer to actual treatment procedures as there were no COVID patients. Instead, it corresponded to the fact that the hospital was on standby for the next wave, having had all existing facilities in place. Infection control costs were progressively larger in terms of percentages as the pandemic evolved.

Panel D shows that in-kind donations from the public constituted a non-negligible part of the hospital’s operations during the pandemic. While, in Phases 1 and 3, they made up only 0.07% and 0.50% of the total costs respectively, they accounted for 13.43% during the pandemic (Phase 2). Donated items included, for example, infrared thermometers, oxygen concentrators, N95 masks, meal boxes, and hotel stays for staff in COVID wards. If public donations had not been provided, the hospital would have had to purchase the donated items and cover 79.06 + 13.43 = 92.49% of the total Phase 2 cost, instead of 79.06%. This was equivalent to the hospital having had to increase its contribution by 13.43/79.06 = 16.98%. Subsidies from the government were also a substantial cost component, making up 7.18% and 7.51% of the total costs during Phases 1–2 respectively.

Finally, Panel E shows direct and indirect costs of the teaching hospital. Direct costs referred to costs that were directly related to care of COVID-19 patients and included diagnostic and treatment procedures. Indirect costs were defined as costs not directly related to care of COVID-19 patients and pertained to all other activities, including, for example, preparation and communications, infection control, and personnel benefits. In other words, the former was a direct function of the number of COVID cases (confirmed or otherwise) received by the hospital, while the latter was not. The distinction between direct and indirect costs here should not be confused with alternative definitions in existing studies [10, 12, 22], where the term ‘indirect costs’ may refer to productivity loss during an outbreak. Panel E corresponds closely to Panel B. The direct cost in Phase 1 was driven primarily by diagnostic procedures and the fact that the hospital renovated some spaces in preparation for the upcoming pandemic, while that in Phase 2 was largely attributable to treatment procedures and the use of spaces for treatment. Finally, the direct cost in Phase 3 was mainly due to the fact that the hospital had its COVID-designated spaces on standby.

Table 3 provides a closer look at the costing process, showing the breakdown by input types. The figures refer to percentages of the total cost in each phase. Summing up items 1.1, 1.3, 1.4, 2.1 and 3.1 in each column yields the percentage of the total cost attributable to treatment in the respective phase, with the rest of the items referring to infection control. In Phase 1, the biggest cost driver was the use of materials related to treatment, accounting for 52.24% of the total cost, followed by labor (15.98%) and capital (13.87%) pertaining to treatment. In Phase 2, a similar pattern was observed, although it could be seen that personnel benefits now constituted a substantial portion of the total cost, accounting for 9.28+1.13 = 10.41% of the total cost. However, as explained earlier, room and board benefits (i.e., hotel stays for staff that were unable to go home because of their close contact with COVID patients, and meal boxes) were entirely donated. Finally, in Phase 3, labor and material costs were all related to infection control, with 0% of such costs being attributed to treatment in the absence of COVID patients. Capital costs for treatment and infection control accounted for 38.03% and 24.12% of the total cost in Phase 3 respectively, replacing materials as the major cost driver.

**Table 3:** Breakdown of cost by type of inputs.

Type of inputs^a^	Study Period		
	Phase 1 ’Preparation’	Phase 2 ’Pandemic’	Phase 3 ’Standby’
**Month**	Jan-Feb 2020	Mar-May 2020	Jun-Oct 2020
1. Labor	19.77%	29.06%	14.84%
1.1. Treatment (+ incentive payments)	15.98%	16.88%	0.00%
1.2 Infection control	3.79%	1.77%	8.91%
1.3 Room and board	0.00%	9.28%	0.00%
1.4 Insurance	0.00%	1.13%	5.93%
2. Material	66.07%	62.29%	23.02%
2.1 Treatment	52.24%	42.75%	0.00%
2.2 Infection control	13.84%	19.54%	23.02%
3. Capital	14.16%	8.65%	62.15%
3.1 Treatment	13.87%	5.20%	38.03%
3.2 Infection control	0.28%	3.45%	24.12%
**Total cost (in USD)** ^b^	605,984.68	3,891,705.94	1,234,424.13

^a^ Figures refer to percentages of the total costs.

^b^ Exchange rate of 1 USD: 30 THB was used.

## Discussion

This study estimated costs that the first wave of COVID-19 imposed on a large teaching hospital in Thailand, separating the 10-month data collection period from January to October 2020 into three phases. The total costs were 605,984.68; 3,891,705.94 and 1,234,424.13 USD for the preparation, the pandemic, and the standby stages, or Phases 1–3, respectively. In Phases 1–2, materials represented the most important cost item, while, for Phase 3, it was capital. Costs related to treatment and screening of patients with suspected and confirmed COVID-19 were considerably higher than infection control costs in Phases 1–2, making up 82.09% and 75.23% of the total costs respectively. However, in Phase 3, the treatment cost was lower than the infection control cost, accounting for 43.95% of the total cost in the absence of COVID-19 patients. The hospital received ad hoc funding from the government in the form of incentive payments for medical staff and from the public in the form of in-kind donations, making up 7.35%, 20.94%, and 0.50% in Phases 1–3 respectively.

To the authors’ knowledge, this study was the first to analyze hospital costs of COVID-19 in Thailand, to perform costing for different phases of the first wave and for different areas in the same hospital, and to assess the importance of in-kind donations during the pandemic. It illustrated using a case study that costs associated with a pandemic were incurred not only during, but also before and after the pandemic. Focusing only on the period in which the pandemic took place would produce an underestimate. The study also demonstrated that contributions from the public were substantial and should be accounted for. For the case study, in-kind donations in the form of medical supplies and equipment were integral to the provision of services especially during the pandemic phase, as medical products had become scarce in the market and the hospital would have had to source and consequently bear expenses for most of the donated items.

Consistent with the literature, this study showed that, to measure the financial impact of an outbreak, costs of both treatment and infection control needed to be accounted for. Existing studies on hospital-level costing demonstrated the importance of infection control activities among medical personnel [13] as well as intra-ward [14, 18, 22] and hospital-wide [12, 15, 16, 19, 23–25] infection control measures during outbreaks. With some exceptions [13, 16, 19], treatment costs were usually covered. Most studies performed micro-costing and reported per-patient costs, using data from either one hospital [12, 14, 15, 18, 19, 22–24] or a selected group of hospitals [9], while others relied on cost and charge data from a national database [10, 11]. Nevertheless, results varied and were not directly comparable, as the setting and the context of the outbreak differed across studies. It is worth noting that published works on costing of outbreak containment in developing countries in general [9] and Thailand in particular [13] are scarce.

Findings in this study should be interpreted with caution. First, albeit consistent with most studies in the literature [12, 14, 15, 18, 19, 22–24], data used in this study came from one hospital and the results were not representative of other hospitals in Thailand. Nevertheless, it was not possible to undertake costing at the national level since there was no database on hospital resources used for COVID-19 in the country. Second, the data were incomplete and potentially subjected to reporting errors. For example, it was not possible to calculate costs for patients with different levels of illness severity since illness severity was inconsistently recorded. Existing studies similarly observed problems of data quality during COVID-19 [9, 11]. Third, the analysis was restricted only to the first wave and did not cover the later and the current waves, where the number of cases was considerably higher and the financial impact was likely to be larger. Results from this study should be thought of as a lower-bound estimate of costs in the later waves.

Finally, this study did not account for foregone revenue due to closure of non-COVID wards and clinics [12, 18, 22, 23, 25]. However, quantifying foregone revenue may not be appropriate in this context. As discussed in the Introduction, most patients at public hospitals in Thailand, including the teaching hospital in question, are covered by a public health insurance program and, with complex provider reimbursement mechanisms, hospitals will receive revenue but may not make ‘profits’ providing treatment, as the reimbursement rate could be lower than the actual cost [4]. Combining costs with foregone revenue due to ward closure during COVID-19 would have produced confusing results, making the hospital’s financial status appear better or worse than it actually was.

This study has broader implications. First, it suggests that resources in the health system be efficiently mobilized and interventions, including most notably a booster dose rollout, be implemented at an accelerated pace, as the COVID-19 pandemic carries substantial costs. Second, this study suggests that hospitals set up a contingency fund for medical emergencies as they seem to be increasingly more likely. The above results could be used as an input toward budget and investment planning for treatment and prevention strategies of COVID-19 itself or pandemics of similar magnitude in the future [10, 11]. They may also be used to determine an appropriate reimbursement rate within the Thai public health insurance system in the presence of a national outbreak. Finally, the difficulty of collecting data for costing purposes is well noted. This study suggests that hospitals develop a single comprehensive database and use a centrally designed template to collect financial and clinical data from different divisions to minimize inconsistencies and ensure data completeness.
